# Gender and age differences in the global burden of peptic ulcers: an analysis based on GBD data from 1990 to 2021

**DOI:** 10.3389/fmed.2025.1586270

**Published:** 2025-04-28

**Authors:** Ruirui Tan, Dan Zhao, Xiaomei Zhang, Tong Liu, Chao Han, Zhongcheng Li, Chenxi Qi, Zhaohui Wang

**Affiliations:** ^1^College of Acupuncture and Massage, Changchun University of Chinese Medicine, Changchun, China; ^2^Department of Acupuncture and Moxibustion, Shenzhen Hospital of Integrated Traditional Chinese and Western Medicine, Shenzhen, China; ^3^Department of Acupuncture and Moxibustion, Shenzhen Bao’an Authentic TCM Therapy Hospital, Shenzhen, China; ^4^Department of Traditional Chinese Medicine, Liaoning University of Traditional Chinese Medicine, Shenyang, China

**Keywords:** global disease burden, peptic ulcer, autoregressive integrated moving average model, join-point regression, age-period-cohort analysis

## Abstract

**Background:**

Peptic ulcer (PU) is one of the most common gastrointestinal diseases worldwide. With advances in medical technology, the global disease burden of PU has been effectively controlled. However, the most recent evidence regarding the global burden of PU remains limited.

**Methods:**

Using publicly available data from the Global Burden of Disease (GBD) study from 1990 to 2021, we analyzed the characteristics of the global burden of PU, including trends in incidence, prevalence, mortality, disability-adjusted life years (DALYs), years lived with disability (YLDs), and years of life lost (YLLs). We employed Joinpoint regression, age-period-cohort (APC) analysis, decomposition analysis, and autoregressive integrated moving average (ARIMA) modeling to evaluate changes and influencing factors for each indicator.

**Results:**

The global number of PU cases increased from 2,570,413 in 1990 (95% CI: 2,161,831–2,997,880) to 2,854,370 in 2021 (95% CI: 2,438,231–3,264,252), representing a cumulative growth of 11.05%. However, the age-standardized incidence rate (ASIR) decreased from 57.14 (95% CI: 48.61–66.73) per 100,000 population in 1990 to 34.10 (95% CI: 29.13–38.97) per 100,000 population in 2021. The global number of deaths due to PU decreased from 273,872 in 1990 (95% CI: 247,312–299,718) to 230,217 in 2021 (95% CI: 193,005–270,858). Significant gender differences were observed, with the disease burden consistently higher in males than in females. After controlling for period and cohort effects, the onset of PU tended to occur at younger ages, and the number of cases declined across most age groups from 1990 to 2021. The highest incidence of PU was concentrated in individuals aged 90 years and older. In terms of future trends, the global incidence of PU is projected to continue decreasing over the next 15 years. The global prevalence is expected to improve, while PU-related mortality is likely to plateau without significant increases or decreases.

**Conclusion:**

The global burden of PU has declined significantly over the past three decades. However, elderly individuals and males remain at higher risk and require continued attention in prevention and management efforts.

## 1 Introduction

Peptic ulcers (PUs) are usually located in the stomach or proximal duodenum but can also be found in the esophagus or Meckel’s diverticulum (1). Globally, approximately 5–10% of individuals will develop a peptic ulcer, with an annual incidence estimated at 0.3–1.9% (2). The condition is caused due to an imbalance between the protective mechanisms and the corrosive effects of gastric acid and pepsin (1). Dysfunction of mucosal defense, coupled with hyperacidity, leads to progressive erosion, ranging from superficial damage to deep ulceration (3). In severe cases, complications such as bleeding, perforation, pyloric obstruction, and even malignancy may occur, significantly impairing patients’ quality of life.

Current treatment strategies for peptic ulcers primarily involve acid-suppressing medications and antimicrobial therapy (4). However, the increasing prevalence of antimicrobial resistance and the growing use of antithrombotic agents in aging populations have added complexity to disease management. The global prevalence of peptic ulcer ranges from 0.12 to 1.5% (3), with estimates in Western populations ranging from 5 to 10% (1). Mortality rates have declined among individuals born in the 20th century compared to those born in the 19th century, as indicated by various epidemiological surveys (5–7). In the United States, hospital admissions related to peptic ulcers decreased by 25.8% between 2005 and 2014, with in-hospital mortality rates declining by 2.4% over the same period (8). A similar downward trend has been observed in Asian populations over the past two decades, suggesting a birth cohort effect in peptic ulcer epidemiology (7).

With increasing global awareness of health and safety, risk factors, disease burden, and advancements in therapeutic technologies are shaping the epidemiological patterns of peptic ulcer. However, monitoring long-term trends in the global burden of the disease remains a challenge due to geographical disparities and the lack of comprehensive global analyses (9). The Global Burden of Diseases, Injuries, and Risk Factors (GBD) study has recently updated its dataset to 2021, incorporating revised estimates across the entire time series and including health statistics from over 200 countries (10). While the GBD primarily provides macro-level assessments of disease epidemiology, a more detailed analysis of peptic ulcer trends is necessary (11).

In this context, the present study examines the global burden of peptic ulcer disease from 1990 to 2021, encompassing prevalence, morbidity, mortality, disability-adjusted life years (DALYs), years of life lost (YLLs), and years lived with disability (YLDs). Additionally, it explores age-specific patterns, gender differences, and temporal trends to provide critical statistical insights for optimizing health policies, healthcare resource allocation, and patient management strategies.

## 2 Materials and methods

### 2.1 Study population

The data used in this study were derived from the GBD database, which was constructed from 46,749 cohort studies, randomized controlled trials, civil surveys, and other research sources. The GBD 2021 report presents data on incidence, prevalence, and mortality rates for 369 diseases and injuries across 204 countries and regions. The present study evaluates global data on the incidence, prevalence, mortality, and DALYs of PU patients from 1990 to 2021. The data were analyzed across different age groups, genders, years, and regions. The DALYs include YLLs, representing the number of years of life lost due to premature mortality, and YLDs, representing the number of years of life lived with disability.

### 2.2 Data collection

In this study, data were obtained from the Global Health Data Exchange Outcomes Tool^1^, which was used to extract disease-related data variables, including 95% confidence intervals, and to analyze the burden of disease across both age-standardized and age-specific rates.

The primary standardization tools employed in this study were Bayesian meta-regression (Dis Mod-MR), spatio-temporal Gaussian process regression (ST-GPR), and autoregressive integrated moving average (ARIMA) models. Dis Mod-MR is a Bayesian meta-regression tool that evaluates all available data on the incidence, prevalence, and mortality of a disease, thereby enhancing consistency between epidemiological parameters.

ST-GPR is used to analyze spatio-temporal data, while the ARIMA model is a classical statistical model used for time series data forecasting to predict future trends in disease incidence, prevalence, and mortality. As GBD data are globally publicly available, no ethics committee approval was required for this study. Furthermore, this study adhered to the standards set forth for accurate and transparent health assessment reports.

### 2.3 Statistical analyses

The incidence, prevalence, mortality, DALYs, YLDs, and YLLs of global PUs were extracted from the GBD database, along with their corresponding age-standardized incidence rates (ASIR), age-standardized prevalence rates (ASPR), and age-standardized mortality rates (ASMR). Furthermore, the dataset includes age-standardized DALY rates (ASDR), age-standardized YLD rates, and age-standardized YLL rates.

To determine trends in disease burden, the corresponding 95% confidence intervals (95% CI) were calculated using the Joinpoint software (National Cancer Institute, Rockville, Maryland, United States of America). Model optimization was conducted using permutation tests with a significance level (alpha) set at 0.05 and 4,499 permutations. The significance of joinpoints was tested using this threshold. The number of joinpoints was set to range from a minimum of 0 to a maximum of 5, and the program determined the optimal number of joinpoints for the model. The model was calculated by estimating the pattern of change in incidence using the least squares method, which avoids the non-objectivity of typical trend analyses based on linear trends. Additionally, the sum of the squares of the residuals between the estimated and actual values was calculated, which yielded the turning point of the moving trend (12). The mean annual percentage change (AAPC) was calculated as 100 × (exp (*β*) - 1), with 95% CI also calculated from the model. If AAPC > 0, the age-standardized metrics demonstrated an increasing trend; if AAPC < 0, a decreasing trend was indicated; and if AAPC = 0, a more stable trend was suggested. To evaluate temporal trends in PU burden, regression analyses were employed. In particular, the regression curves were transformed into the natural logarithm of the age-standardized rate (ASR) using the following equation: y = a + bx + £, where y represents the natural logarithm of the ASR, x denotes the calendar year, and £ represents the error term. To fit age-standardized indicators to the regression model, the following equation is used: ln (y) = *α* + βx + *ε*, where y represents the corresponding age-standardized indicator and x represents the calendar year. All rates are reported as mean estimates per 100,000 population, which are determined by ranking the 25th and 75th values in the ranked 1,000 draws (13).

To examine the effects of age, period, and cohort on health outcomes, the study employed the use of age-period-cohort (APC) modeling (14). The term ‘age effect’ refers to the risk of an outcome occurring at different ages. The period effect represents the impact of temporal changes on outcomes observed across all age groups. Cohort effects pertain to changes in outcomes experienced by individuals from the same birth cohort (15). The APC model was fitted using the Epi package (version 2.46) in R (version 4.2.0)^2^. The residual deviations between the models and the AIC were compared to determine the optimal model. A decomposition analysis was employed to ascertain the influence of each factor (three cohort-level determinants: population aging, population growth, and epidemiological change) on the overall change (16–18).

The ARIMA model comprises an autoregressive model (AR) and a moving average (MA) model. The fundamental tenets of the ARIMA model are that the data series are time-dependent random variables and that the autocorrelation can be characterized by the ARIMA model. Furthermore, the model posits that future values can be predicted based on past values. The equation is expressed as *Y_t_ = _φ1_Y_t-1_ + _φ2_Y_t-2_ +…_φp_Y_t–p_ + e_t_-θ_1_e_t-1_-…-θ_q_ e_t-q_* (19), where (*_φ1_Y_t-1_ + _φ2_Y_t-2_ +…_φp_Y_t_ + e_t_*) is the AR model part, *e_t_-θ_1_e_t-1_-…-θ_q_ e_t-q_* is the MA model part, Y*_t–p_* is the observed value at the period of (*t-p*), p and q represent the model order of AR and MA, and *e_t_* is the random error at the period of *t.* In the ARIMA modeling process, differencing is first applied to stabilize the time series. The *auto.arima ()* function is then used to select the optimal model based on the Akaike Information Criterion (AIC) (20). To assess the residuals, we employ residual plots, autocorrelation function (ACF) plots, and residual Q-Q plots to check for normality. Finally, the Ljung-Box test is conducted to determine whether the residuals constitute white noise.

The data from this study were subjected to statistical analysis and visualization using the R statistical software program (version 4.4.1) and the Joinpoint software program (version 5.2.0). A *p*-value of less than 0.05 was considered to be statistically significant.

## 3 Results

### 3.1 Descriptive analysis of PU burden globally

#### 3.1.1 Incidence of PU globally

The global number of PU cases increased from 2,570,413 in 1990 (95% CI: 2161831–2,997,880) to 2,854,370 in 2021 (95% CI: 2,438,231–3,264,252), representing a cumulative growth of 11.05%. However, the ASIR globally decreased from 57.14 (95% CI:48.61–66.73) per 100,000 population in 1990 to 34.10 (95% CI:29.13–38.97) per 100,000 population in 2021. Meanwhile, the AAPC of the global incidence rate decreased by 1.65% (95% CI: 1.69–1.61) from 1990 to 2021 (Table 1). According to gender analysis, from 1990 to 2021, the number of incidence cases in men and the ASIR per 100,000 population both declined but remained higher in women (Table 2).

**Table 1:** All-age cases and age-standardized incidence, prevalence, deaths, DALYs, YLDs, and YLLs rates of PU in globally in 1990 and 2021 by total.

Location	Measure	1990		2021		AAPC, *n* (95% CI)
		All-age cases	Age-standardized rates per 100,000 people	All-age cases	Age-standardized rates per 100,000 people	
		*n* (95% CI)	*n* (95% CI)	*n* (95% CI)	*n* (95% CI)	
Global	Incidence	2,570,413 (2,161,831–2,997,880)	57.14 (48.61–66.73)	2,854,370 (2,438,231–3,264,252)	34.10 (29.13–38.97)	−1.65 (−1.69–1.61)
	Prevalence	6,038,112 (5,268,704–7,064,284)	132.97 (116.22–154.17)	6,567,782 (5,798,379–7,597,596)	78.27 (69.02–90.76)	−1.69 (−1.74–1.63)
	Deaths	273,872 (247,312–299,718)	7.14 (6.41–7.82)	230,217 (193,005–270,858)	2.75 (2.31–3.24)	−3.02 (−3.13–2.91)
	DALYs	8,394,780 (7,603,519–9,210,479)	193.82 (175.79–212.14)	6,057,594 (5,162,099–7,041,854)	71.56 (61.07–83.05)	−3.17 (−3.24–3.10)
	YLDs	310,547 (207,881–428,151)	6.79 (4.58–9.36)	334,884 (225,914–467,689)	4.00 (2.70–5.59)	/
	YLLs	8,084,233 (7,276,895–8,855,839)	187.03 (168.46–204.58)	5,722,709 (4,838,577–6,693,440)	67.56 (57.14–78.90)	

DALYs, Disability-Adjusted Life Years; YLLs, Years of Life Lost; YLDs, Years Lived with Disability.

**Table 2:** All-age cases and age-standardized incidence, prevalence, mortality, DALYs, YLDs, and YLLs rates of PU in globally in 1990 and 2021 by gender.

Location	Measure	1990				2021				AAPC	
		All-ages cases		Age-standardized rates per 100,000 people		All-ages cases		Age-standardized rates per 100,000 people			
		Females	Males	Females	Males	Females	Males	Females	Males	Females	Males
		*n* (95% CI)	*n* (95% CI)	*n* (95% CI)	*n* (95% CI)	*n* (95% CI)	*n* (95% CI)	*n* (95% CI)	*n* (95% CI)	*n* (95% CI)	*n* (95% CI)
Global	Incidence	1,122,572 (953,306–1,299,689)	1,447,840 (1,208,065–1,696,238)	48.13 (41.11–56.00)	66.53 (56.42–77.67)	1,327,659 (1,140,550–1,515,872)	1,526,711 (1,298,990–1,752,373)	30.88 (26.36–35.41)	37.45 (32.04–42.73)	−1.42 (−1.46 – −1.37)	−1.84 (−1.88 – −1.81)
	Prevalence	2,647,534 (2,303,521–3,109,910)	3,390,578 (2,958,938–3,963,070)	112.44 (98.04–130.80)	153.970 (134.15–178.5)	3,054,357 (2,693,131–3,534,977)	3,513,425 (3,101,421–4,067,670)	71.138 (62.30–82.63)	85.70 (75.66–99.22)	−1.46 (−1.50– −1.41)	−1.87 (−1.91–1.84)
	Deaths	107,766 (93,428–124,017)	166,106 (146,750–187,305)	5.20 (4.51–5.96)	9.44 (8.36–10.69)	103,950 (84,951–123,377)	126,267 (10,5,491–151,015)	2.26 (1.85–2.68)	3.31 (2.76–3.95)	−2.63 (−2.75–2.51)	−3.33 (−3.42–3.23)
	DALYs	3,073,203 (2,665,598–3,561,820)	5,321,577 (4,675,648–5,960,577)	135.46 (117.62–155.86)	257.033 (226.68–288.22)	2,521,948 (2,123,545–2,946,161)	3,535,645 (2,982,846–4,223,698)	57.052 (48.21–66.30)	87.02 (73.46–103.85)	−2.76 (−2.82–2.69)	−3.45 (−3.53–3.37)
	YLDs	145,472 (98,316–202,119)	165,075 (110,567–228,500)	6.12 (4.14–8.45)	7.49 (5.05–10.32)	166,882 (113,840–232,086)	168,002 (112,886–234,197)	3.91 (2.67–5.44)	4.11 (2.76–5.73)	/	
	YLLs	2,927,731 (2,535,317–3,442,397)	5,156,503 (4,501,418–5,772,336)	129.34 (111.62–150.65)	249.54 (219.29–280.27)	2,355,066 (1,966,675–2,765,087)	3,367,643 (2,821,321–4,051,243)	53.14 (44.61–62.18)	82.91 (69.54–99.39)		

DALYs, Disability-Adjusted Life Years; YLLs, Years of Life Lost; YLDs, Years Lived with Disability; AAPC, average annual percentage change.

#### 3.1.2 Prevalence of PU globally

In terms of prevalence, the number of PU cases increased from 603,811,2 in 1990 (95% CI: 5,268,704–7,064,284) to 6,567,782 in 2021 (95% CI: 5,798,379–7,597,596), representing a cumulative growth of 8.77%. However, the ASPR decreased from 132.97 (95%CI: 116.22–154.17) per 100,000 population in 1990 to 78.27 (95%CI: 69.02–90.76) per 100,000 population in 2021. The AAPC of the mortality rate globally decreased by 1.69% (95% CI: 1.74–1.63) from 1990 to 2021 (Table 1). According to gender analysis, from 1990 to 2021, the number of prevalence cases in men and the ASPR per 100,000 population both declined but remained higher in women (Table 2).

#### 3.1.3 Mortality of PUs globally

The number of PU cases globally has decreased from 273,872 in 1990 (95% CI: 247,312–299,718) to 230,217 in 2021 (95% CI: 193,005–270,858). The age-standardized mortality rate (ASMR) decreased from 7.14 (95% CI: 6.41–7.82) per 100,000 population in 1990 to 2.75 (95% CI: 2.31–3.24) per 100,000 population in 2021. The AAPC of the mortality rate globally decreased by 3.02% (95% CI: 3.13–2.91) from 1990 to 2021 (Table 1). According to gender analysis, from 1990 to 2021, the number of mortality cases in men and the ASMR per 100,000 population both declined but remained higher in women (Table 2).

#### 3.1.4 DALYs of PUs globally

In terms of DALYs (including YLDs and YLLs), the number of PU cases decreased from 8,394,780 in 1990 (95% CI: 7,603,519–9,210,479) to 6,057,594 in 2021 (95% CI: 5,162,099–7,041,854), representing a cumulative decrease of 27.84%. However, the ASDR decreased from 193.82 (95%CI: 175.79–212.14) per 100,000 population in 1990 to 71.56 (95%CI: 61.07–83.05) per 100,000 population in 2021. The AAPC of the DALYs rate globally decreased by 3.17% (95% CI: 3.24–3.10) from 1990 to 2021 (Table 1). According to gender analysis, from 1990 to 2021, the number of DALYs cases in men and the ASDR per 100,000 population both declined but remained higher in women (Table 2).

### 3.2 Joinpoint regression analysis of PU burden globally

**Figure 1:**
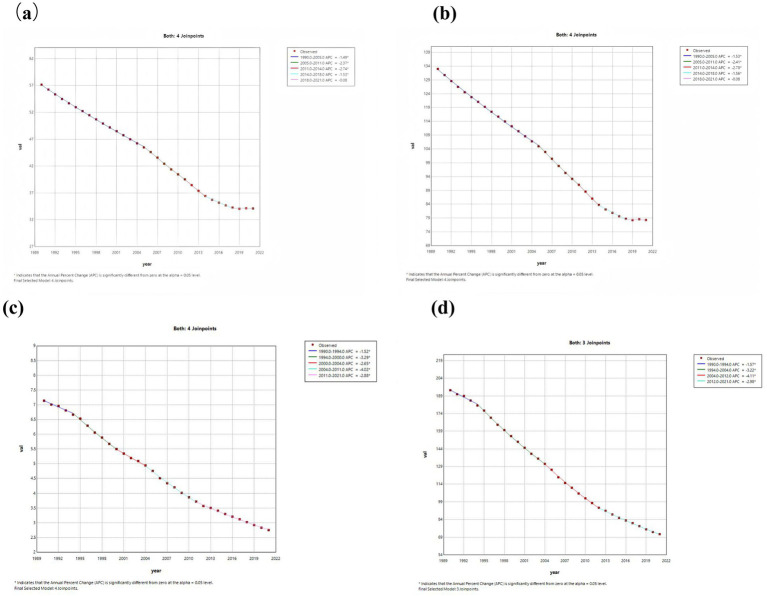
The APC of ASIR, ASPR, ASMR, and ASDR of PU in Global from 1990 to 2021 (*means *p*-values<0.05 and significant results). **(a)** ASIR; **(b)** ASPR; **(c)** ASMR; **(d)** ASDR.

The Joinpoint regression analysis of ASIR, ASPR, ASMR, and DALYs for PU worldwide from 1990 to 2021 is shown in Figure 1. From 1990 to 2018, the ASIR and ASPR showed a significant downward trend globally, while the trend from 2018 to 2021 remained stable. There was no statistically significant difference in APC (*p* > 0.05), as shown in Figures 1a,b. From 1990 to 2021, both ASMR and ASDR globally showed a downward trend, and APC had significant statistical significance (*p <* 0.05), as shown in Figures 1c,d.

### 3.3 Trends in the burden of PU disease globally

**Figure 2:**
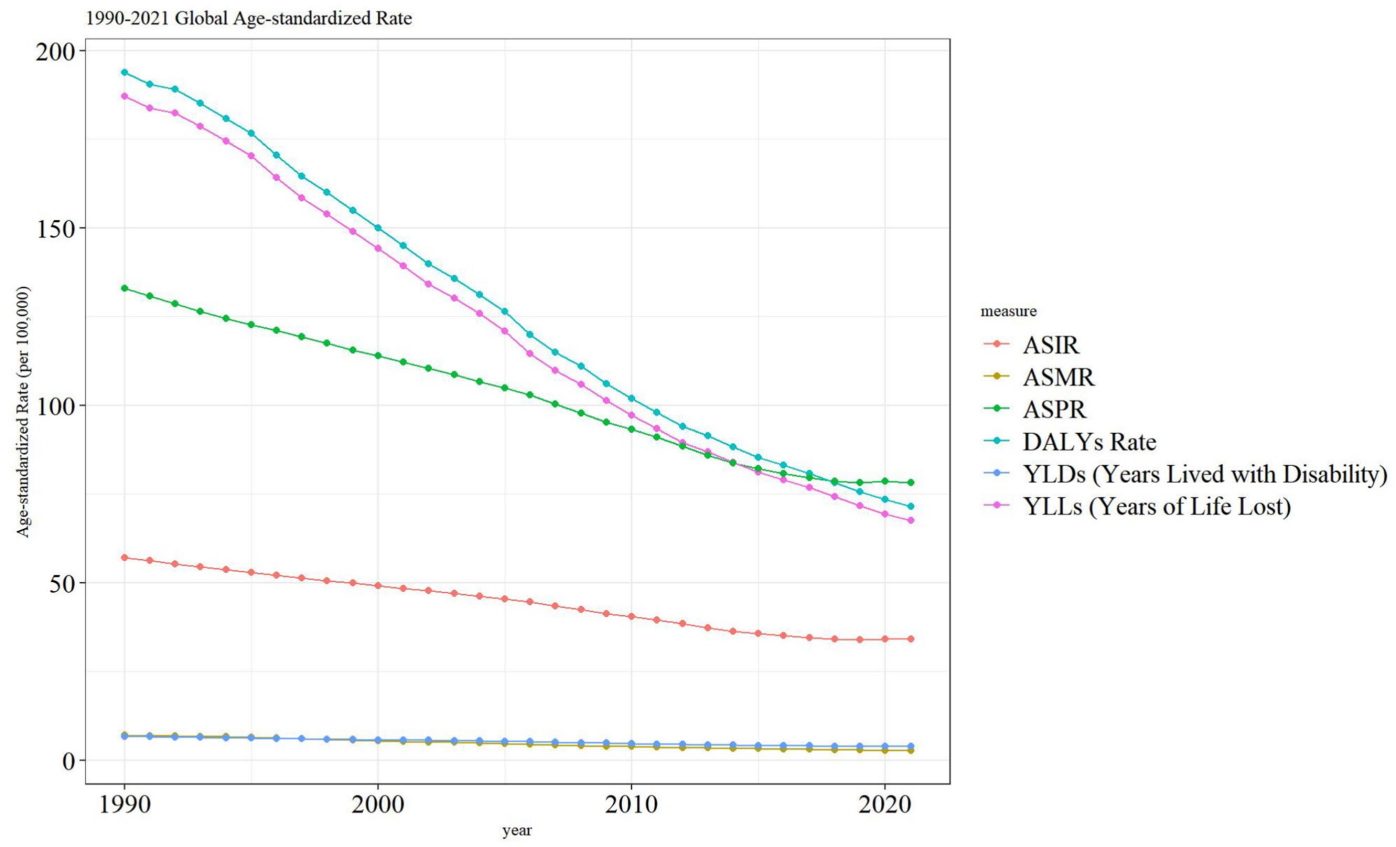
Trend comparison of ASIR, ASMR, ASPR, DALYs, YLDs and YLLs of PU in Global from 1990 to 2021.

The ASDR and YLLs of PU worldwide have gradually decreased from 1990 to 2021. However, the ASIR, ASPR, ASMR, and YSLs of PU have shown a steady trend, as illustrated in Figure 2.

### 3.4 Impact of age-period-cohort on the incidence of PU globally

**Figure 3:**
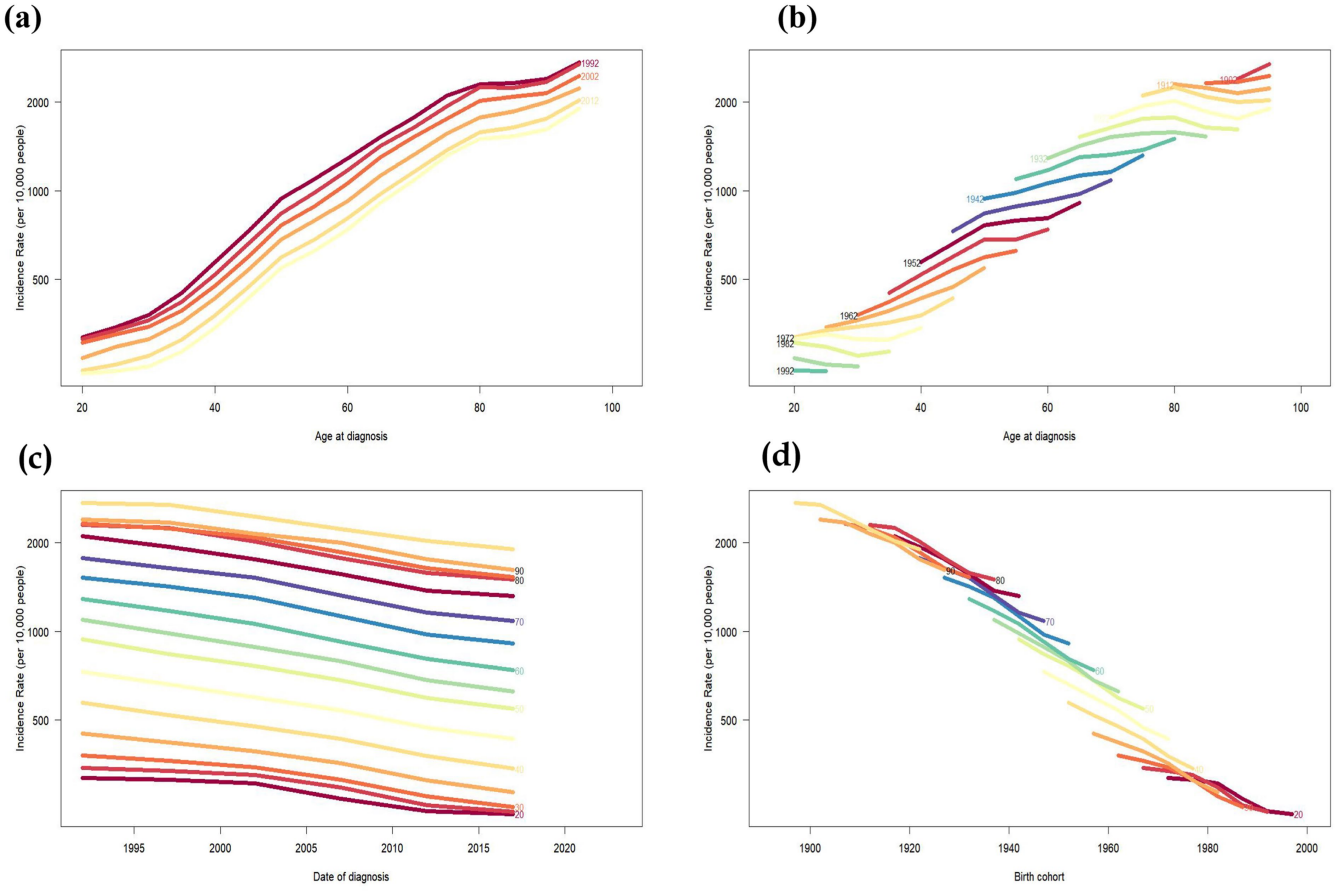
Incidence rates of PU in Global. **(a)** The age-specific incidences rates of PU according to time periods, each line connects the age-specific incidence for a 5-year period. **(b)** The age-specific incidences rates of PU according to birth cohort, each line connects the age-specific incidence for a 5-year cohort. **(c)** The period-specific incidence rates of PU according to age groups, each line connects the birth cohort-specific incidence for a 5-year age group. **(d)** The birth cohort-specific incidence rates of PU according to age groups, each line connects the birth cohort-specific incidence for a 5-year age group.

The time period was divided into 5-year intervals: 1992, 1997, 2002, 2007, 2012, and 2017. Globally, PU was first observed in individuals at the age of 20 years. Over time, the global cohort of individuals with PU showed an upward trend until death, as shown in Figure 3a. After controlling for period and cohort, the onset of PU tended to be younger, and the number of cases decreased in different age groups between 1990 and 2021, as shown in Figure 3b. Globally, the highest number of PU incidence was concentrated in the age group of 90 years and above; over time, the decreasing trend of PU incidence tended to stabilize after 2012 (refer Figure 3c). The results of changes in incidence rates according to age-specific cohorts show that the number of people with incidence rates in each age group has declined over time, as shown in Figure 3d.

### 3.5 Decomposition analysis of PU burden

**Figure 4:**
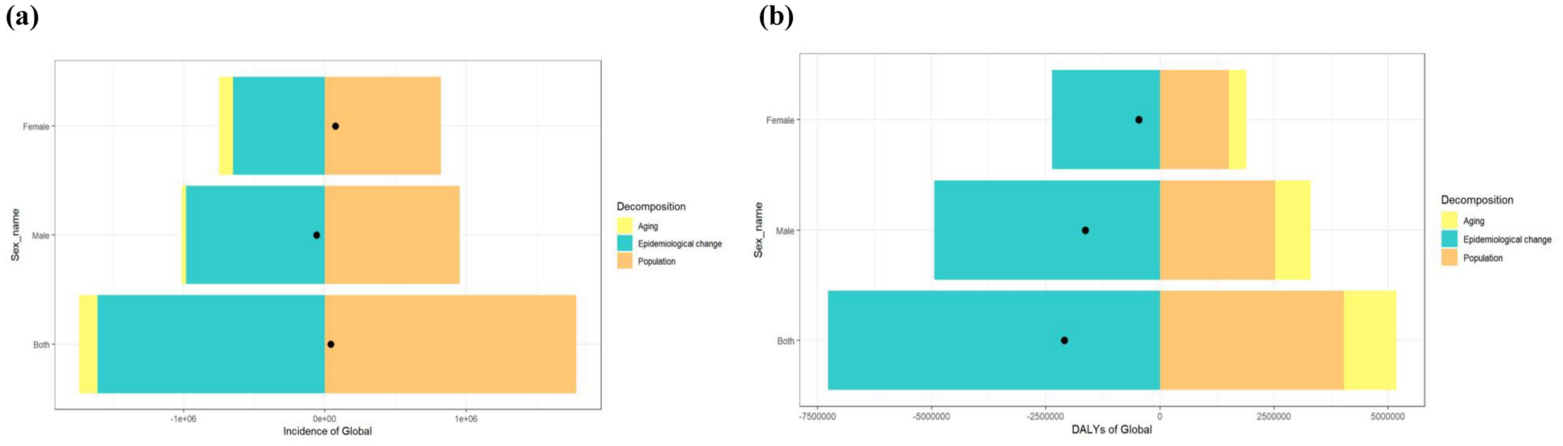
Decomposition analysis of PU indicators from 1990 to 2021. **(a)** Incidence of Global **(b)** DALYs of Global. Black dots represent the overall changes in population growth, aging, and epidemiological changes.

Decomposition analyses showed that aging played a significant role in the PU incidence of men globally (52.75%). Epidemiological changes contributed the most to the global PU DALYs of women globally (504.3%); population growth contributed significantly to global PU incidence,with percentages of 4285.53% (male), -1622.89% (female), and 1114.37% (both sexes combined). The impact of epidemiological changes on global PU incidence was 1670.14% in men and 301.54% in DALYs. Epidemiological changes are the main drivers of changes in PU incidence and DALYs indicators (Supplementary Table 1; Figure 4).

### 3.6 Projections of PU incidence, prevalence, and mortality for the next 15 years

**Figure 5:**
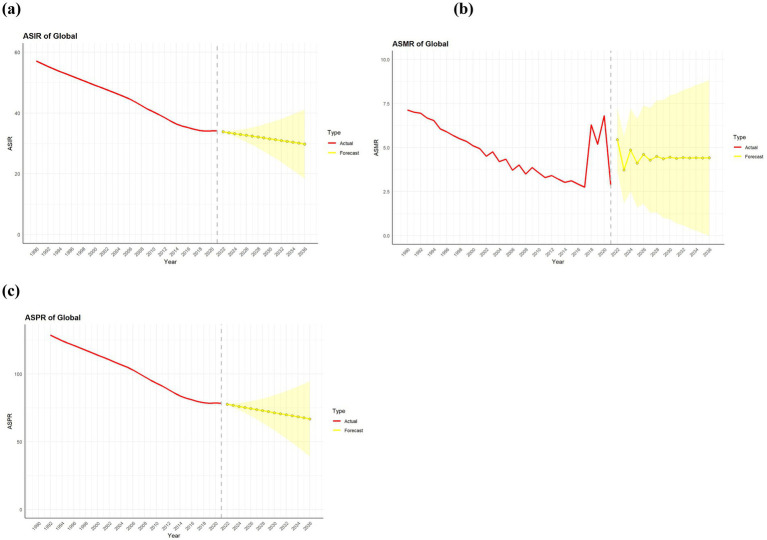
Predicted trends of PU incidence prevalence and death rate in global over the next 15 years (2022–2036). Red lines represent the true trend of PU incidence prevalence and death rate during 1990–2021; yellow dot lines and shaded regions represent the predicted trend and its 95% CI. **(a)** ASIR of Global; **(b)** ASMR of Global; **(c)** ASPR of Global.

The ARIMA model was used to quantitatively describe trends in PU incidence, prevalence, and mortality over the next 15 years. We projected the age-specific burden of PU for the global population. In terms of incidence, the global population of PU is likely to continue decreasing over the next 15 years. The prevalence of PU will improve globally, and the mortality rate for PU will level off over the next 15 years, showing no significant rise or fall, as shown in Figures 5a–c. The optimized model selection for PU incidence was ARIMA (0,2,1), with an AIC value of −55.8 after filtering using the *auto.arima ()* function. Comprehensive diagnostics confirmed that the residuals of the ARIMA (0,2,1) model met the white noise criteria (Ljung-Box Q* = 9.206, df = 5, *p* = 0.101), and no obvious model specification errors were observed in the diagnostic plots (Supplementary Figures 1, 2). Therefore, the model was deemed suitable for predictive analysis. Following the same procedure, we developed the ARIMA (0,2,1) model for PU prevalence (AIC = 9.29), with the Ljung-Box test also confirming its robustness (*χ*^2^ = 12.46, *p* = 0.256). Similarly, the ARIMA (1,1,0) model was selected for PU mortality (AIC = 86.09), and the Ljung-Box test further validated its robustness (*χ*^2^ = 6.002, df = 10, *p* = 0.815). The AIC, BIC, and AICC values of the model (Supplementary Table 2) indicate that the model strikes a good balance between complexity and fitting effectiveness with moderate complexity, making it a candidate model worthy of consideration, while the correlation coefficients of the model and the training set error measurements further validate the reliability of its predictive ability (20, 21).

## 4 Discussion

This study comprehensively assessed the global disease burden and its spatial and temporal trends of PU globally over the past three decades, based on the latest updated GBD 2024 database. It also compared the differences in data by age and gender between 1990 and 2021. The results showed that the global disease burden of PU has significantly decreased, which is consistent with previous findings that the global age-standardized incidence, prevalence, DALYs, and death rates of PU exhibited a decreasing trend. Meanwhile, the number of prevalent cases and the age-standardized prevalence rate were higher in men than in women in all years from 1990 to 2019; the difference between the two groups decreased over time (11).

However, this study has made new and different discoveries. After controlling for period and cohort effects, the onset of PU tended at younger ages, and the number of cases decreased across different age groups between 1990 and 2021. The highest number of PU incidence was concentrated in the age group of 90 years and above. PU appeared at the age of 20 years; the global cohort of individuals with PU was in an upward direction over time until death. The decomposition analysis results of this study indicate that aging played a significant role in the global PU incidence among men (52.75%). In contrast, epidemiological changes were the main contributors to global PU DALYs among women (504.3%). Overall, epidemiological changes emerged as the main drivers of changes in PU incidence and DALYs indicators.

Over the past three decades, the global disease burden of PU has been effectively controlled with the improvement of the global economic standard of living, the spread of people’s awareness of healthcare, and the continuous development of new diagnostic and treatment technologies. The decline in Age-Standardized Incidence Rate and Age-Standardized Prevalence Rate can be attributed to better hygiene, healthier lifestyles, and improved dietary habits. These factors have reduced key risk factors, such as *Helicobacter pylori* infection, which impairs mucosal defense mechanisms (22), thereby contributing to effective disease prevention. In addition, the development of acid-suppressing drugs, such as proton pump inhibitors (23), histamine-2 receptor antagonists (24), Vonoprazan (25), and cellular agents (26), and the advancement of new diagnostic and therapeutic technologies, such as fecal antigen detection (27), endoscopy (28), and adipose mesenchymal stem cells (29), have improved the healthcare management system for PU, enhanced the quality of healthcare services, and improved the prognostic efficacy for patients with PU, thus reducing the ASMR and ASDR of PU patients.

Nevertheless, we must consider the potential threat of global disruptions, such as the COVID-19 pandemic, to the improving trend of PU. For instance, the strain on healthcare resources during the pandemic may have affected the detection and eradication of *Helicobacter pylori* infection, while delays in diagnosis and treatment could have prolonged the disease course or increased complications of PU. Additionally, lifestyle changes during the pandemic, such as shifts in dietary patterns and fluctuations in psychological stress, may have influenced the incidence and prevalence of PU. Recent research evidence indicates that during the pandemic, hospital admissions and mortality rates for peptic ulcer bleeding decreased by approximately 36.6%, while hospital admissions for peptic ulcer perforation dropped by one-seventh. However, mortality rates significantly increased during the pandemic (30). Moreover, in the long term, a significant association has been observed between COVID-19 and an increased risk of gastrointestinal diseases. Even among non-hospitalized COVID-19 patients, the risk of various gastrointestinal disorders, including PU, remains evident, with the risk increasing progressively with the severity of COVID-19 (31). The mechanisms underlying this association remain unclear, but several possibilities have been proposed. One potential explanation is the fecal-oral transmission of the virus, leading to gastrointestinal infection (32). Additionally, interactions between the SARS-CoV-2 spike protein and the expression of the angiotensin-converting enzyme 2 (ACE2) receptor in the digestive tract may contribute to the progression of digestive diseases in COVID-19 patients (33). The detection of SARS-CoV-2 ribonucleic acid (RNA) and intracellular staining of viral nucleocapsid protein in gastric and duodenal epithelium further suggest that these gastrointestinal glandular epithelial cells are infected by SARS-CoV-2 (34). Another possible mechanism involves the significant elevation of inflammatory markers and cytokines, including interleukin-1 (IL-1), IL-6, and tumor necrosis factor-*α* (TNF-α) (35), which play a role in the infiltration of immune cells in the gastrointestinal tract. Therefore, future research should integrate longitudinal data and causal inference methods to quantify the long-term impact of COVID-19 on the burden of PU and explore potential intervention strategies to gain a more comprehensive understanding of the consequences of this global public health event.

Population aging is a major factor influencing the global burden of PU disease. Declining fertility and increasing life expectancy have led to an annual increase in the proportion of older people and an inverted pyramid shift in the demographic structure of society (36, 37). As far as we currently know, the biological mechanisms of PU involve a multifactorial interplay, including an imbalance between mucosal defense and aggressive factors, *Helicobacter pylori* (*H. pylori*) infection, and the use of non-steroidal anti-inflammatory drugs (NSAIDs) (1). In older adults, the risk of ulcer development is increased due to diminished mucosal repair capacity, reduced gastric mucus secretion, and decreased gastric blood flow, combined with a greater susceptibility to the widespread use of NSAIDs (38). Additionally, due to the very different levels of sex hormones in men and women, estrogen can regulate the secretion of basal acid and duodenal bicarbonate and enhance the mucosal barrier function of the gastrointestinal tract by increasing the permeability of epithelial cells and clearing the expression of Erβ mRNA in the nucleus (39). Moreover, gender-related differences in lifestyle habits may play an important role in the development of the disease. The differences in lifestyle habits associated with gender are also important factors influencing disease progression (40). Smoking, alcohol consumption, lack of exercise, and unhealthy diets are all lifestyle habits driven by men’s roles in today’s society (40). Therefore, men are expected to be at a greater risk for PU, which also increases the accuracy and credibility of the results of the present study in predicting the future development of PU.

Therefore, in response to the current risk trends in the disease burden of peptic ulcer, it is necessary for each country and region to take effective countermeasures to control the deterioration of the situation in the future. In particular, as population aging is a globally recognized social problem, it is important to reduce the negative impact of the disease burden by popularizing health knowledge and healthy lifestyles, strengthening health education and awareness of preventive healthcare for the elderly, developing geriatric medical and rehabilitation care services, and improving social security and elderly care systems, thus helping the elderly to have access to a wider range of healthcare services. In addition, the media, educational institutions, and community activities have been used to enhance health knowledge about digestive diseases among men, raise men’s awareness of the dangers of unhealthy lifestyle habits, and formulate and restrict policies to regulate the promotion of cigarettes and alcohol, thereby reducing the threat posed by harmful products to men’s health. In view of the occupational health risks men may face during work, strengthening their occupational health supervision and providing appropriate health examinations and preventive measures can help improve their unhealthy living habits and enhance their overall health.

### 4.1 Strengths and limitations

To the best of our knowledge, this study represents the first comprehensive analysis of the global burden of peptic ulcer disease (PU) from 1990 to 2021, using the latest survey data from the Global Burden of Disease (GBD) study. Additionally, it explores variations in disease burden among PU patients globally based on age and gender. The findings of this study not only provide valuable data to support the global healthcare system but also contribute to the rational allocation of healthcare resources and the advancement of public health management. However, several limitations must be acknowledged. First, the accuracy of our results depends on the quality of the data updated by the GBD in 2021, as well as the completeness, accuracy, and reliability of the standardized models used to optimize these data. Since the GBD study is part of a large international collaborative effort to quantify global health trends, it relies heavily on survey data provided by individual countries (e.g., differences in population structure, unequal distribution of medical resources, and variations in reporting standards across countries), which may be subject to collection and reporting biases. Second, the impact of COVID-19 on PU has not been fully accounted for in this study, potentially leading to an incomplete risk assessment. Future research should incorporate more comprehensive evaluations of the pandemic’s influence on PU incidence, prevalence, and disease burden. Finally, this study focuses only on global prevalence, and more comprehensive research is needed to investigate specific trends in different regions or countries. Additionally, this study did not explore the epidemiological patterns of different types of peptic ulcers. Future research may need to incorporate more granular data sources to further investigate the potential differences between gastric ulcers and duodenal ulcers.

## 5 Conclusion

The global disease burden of PU has declined significantly, with age-standardized incidence, prevalence, mortality, and disability-adjusted life expectancy all being significantly controlled. However, varying trends persist due to the effects of population aging and gender differences. These findings are consistent with the projections of PU trends over the next 15 years in this study. Therefore, timely monitoring of disease prevalence and trend prediction is an important part of disease prevention and control. It is hoped that the results of this study will help explore the characteristics of different disease distribution trends in the future and help local governments formulate better and more detailed policies and interventions according to the characteristics of the diseases.

## Data Availability

The original contributions presented in the study are included in the article/Supplementary material, further inquiries can be directed to the corresponding author.

## Footnotes

^1^ https://vizhub.healthdata.org/gbd-results/

^2^ http://www.r-project.org
