# Supplementary material for: Gender and age differences in the global burden of peptic ulcers: an analysis based on GBD data from 1990 to 2021
Source: Front Med (Lausanne). 2025 Apr 28;12:1586270. doi: 10.3389/fmed.2025.1586270 (PMC12066501; doi:10.3389/fmed.2025.1586270)
Supplement: Supplementary file 1 [file Presentation_1.ZIP › suppl.materials/Suppl Tables.docx]

**Supplementary Tables**

Supplementary table 1. Changes in incidence and DALYs of PU at global levels from 1990 to 2021,decomposed by three-population-level determinants: population aging, population growth and epidemiological changes

| Location | Overall  Difference | Both | | |  | Females | | |  | Males | | |
| --- | --- | --- | --- | --- | --- | --- | --- | --- | --- | --- | --- | --- |
|  |  | Aging  (Percent%) | Population  (Percent%) | Epidemiological change  (Percent  %) |  | Aging  (Percent%) | Population  (Percent%) | Epidemiological change  (Percent  %) |  | Aging  (Percent%) | Population  (Percent%) | Epidemiological  change  (Percent  %) |
| Global |  |  |  |  |  |  |  |  |  |  |  |  |
| Incidence | 41526.11 | -130266.61  (-313.70) | 1779614.2  (4285.53) | -1607821.5  (-3871.83) |  | -97642.45  (-132.54) | 820935.1  (1114.37) | -649624.8  (-881.83) |  | -31020.77  (52.75) | 954356.9  (-1622.89) | -982141.9  (1670.14) |
| DALYs | -2098117.6 | 1143318.8  (-54.49) | 4030840  (-192.12) | -7272277  (346.61) |  | 388106.1  (-82.80) | 1506926  (-321.49) | -2363756  (504.30) |  | 779257.8  (-47.57) | 2522282  (-153.97) | -4939660  (301.54) |

Notes: DALYs: Disability-Adjusted Life Years.

Supplementary table 2. Parameter estimation values and information of ARIMA model (2022-2036).

|  | ASIR | ASPR | ASMR |
| --- | --- | --- | --- |
|  | Global | Global | Global |
| AIC | -55.8 | 9.29 | 86.09 |
| AICc | -55.35 | 9.74 | 86.51 |
| BIC | -52.99 | 12.1 | 88.95 |

Notes: AIC: Akaike Information Criterion; AICc: Corrected Akaike Information Criterion;

BIC: Akaike Information Criterion
