# Supplementary material for: Gender and age differences in the global burden of peptic ulcers: an analysis based on GBD data from 1990 to 2021
Source: Front Med (Lausanne). 2025 Apr 28;12:1586270. doi: 10.3389/fmed.2025.1586270 (PMC12066501; doi:10.3389/fmed.2025.1586270)
Supplement: Supplementary file 1 [file Presentation_1.ZIP › suppl.materials/Suppl.image.docx]

**Supplementary Figures**

**a b**

**
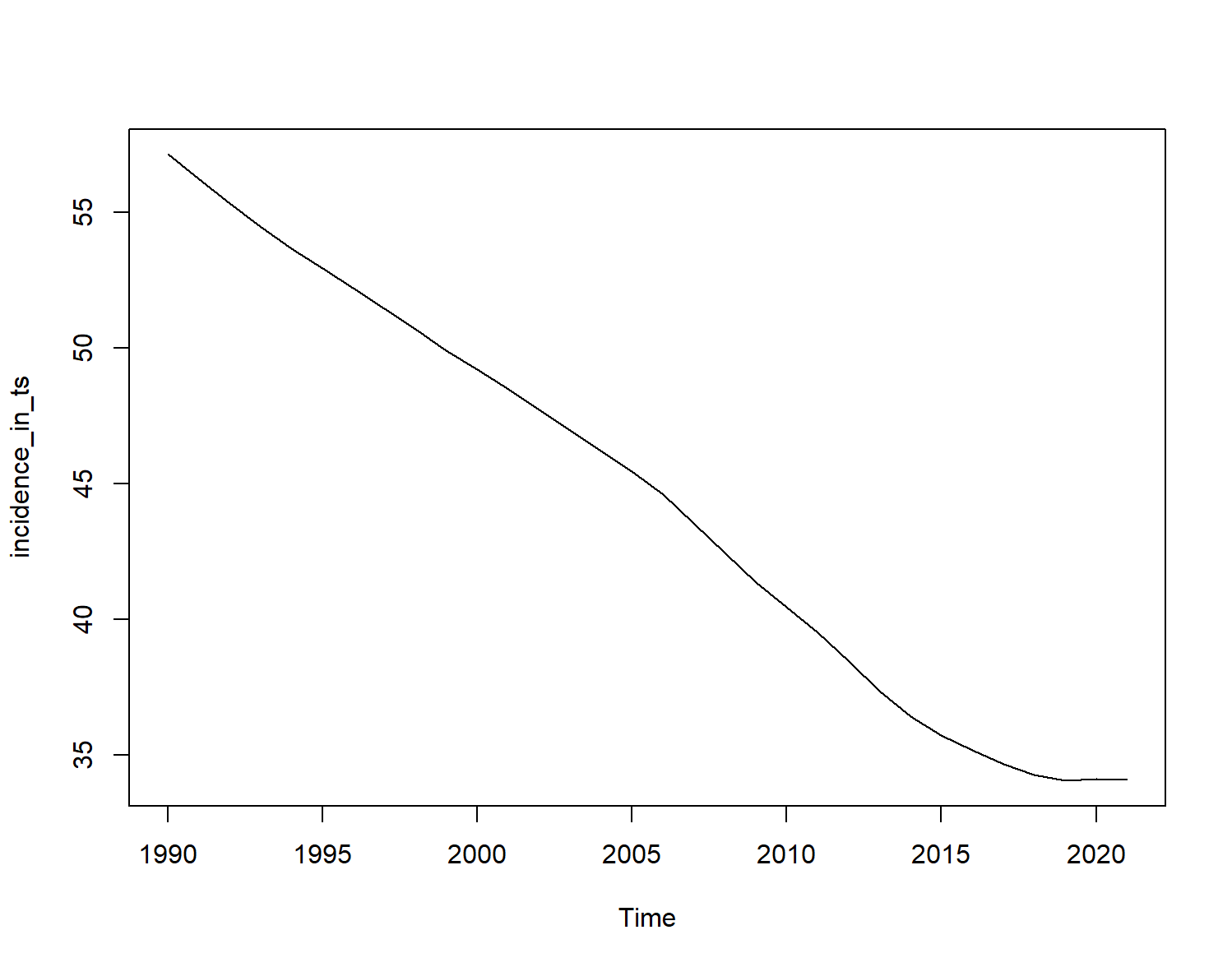

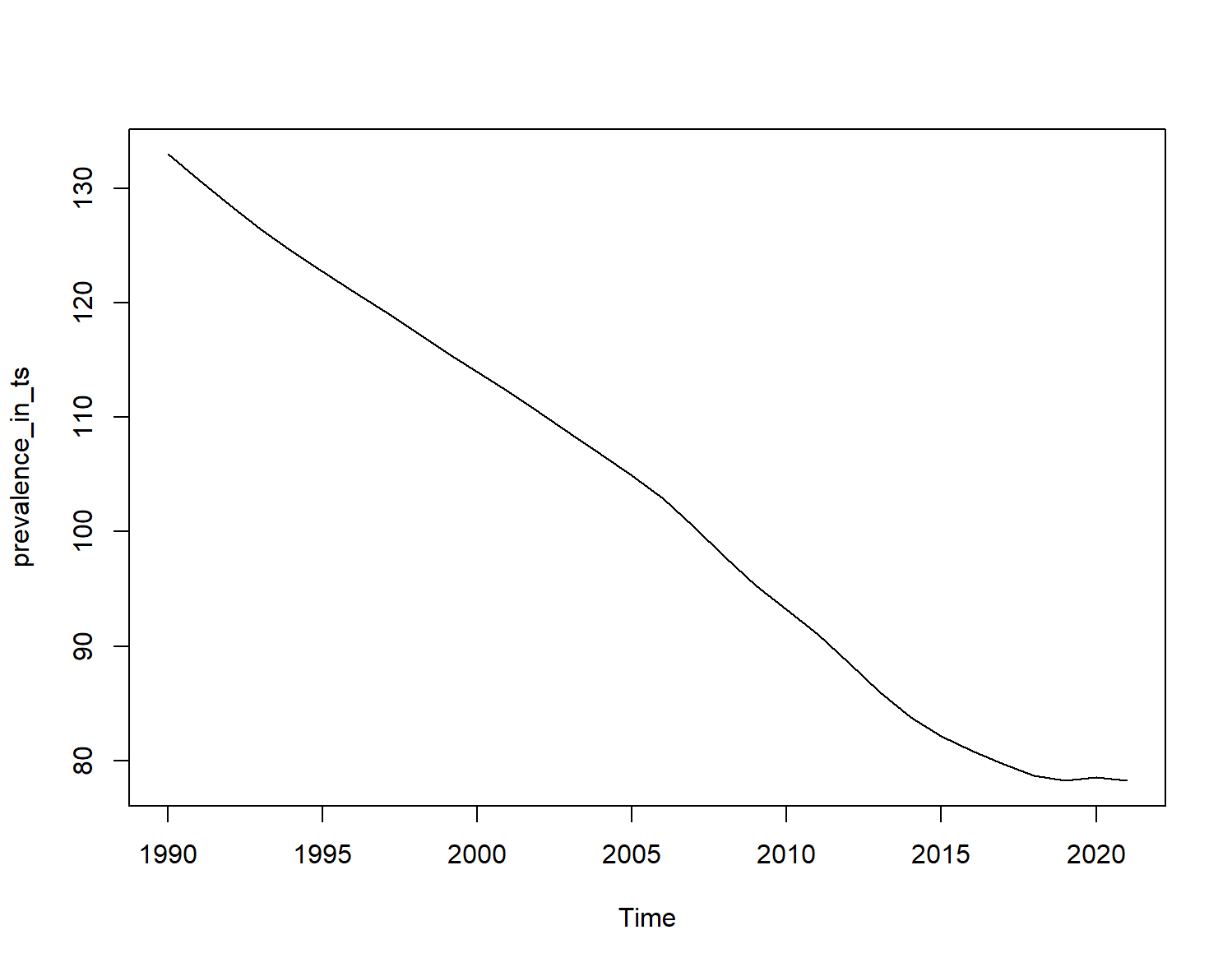
**

**c**

**
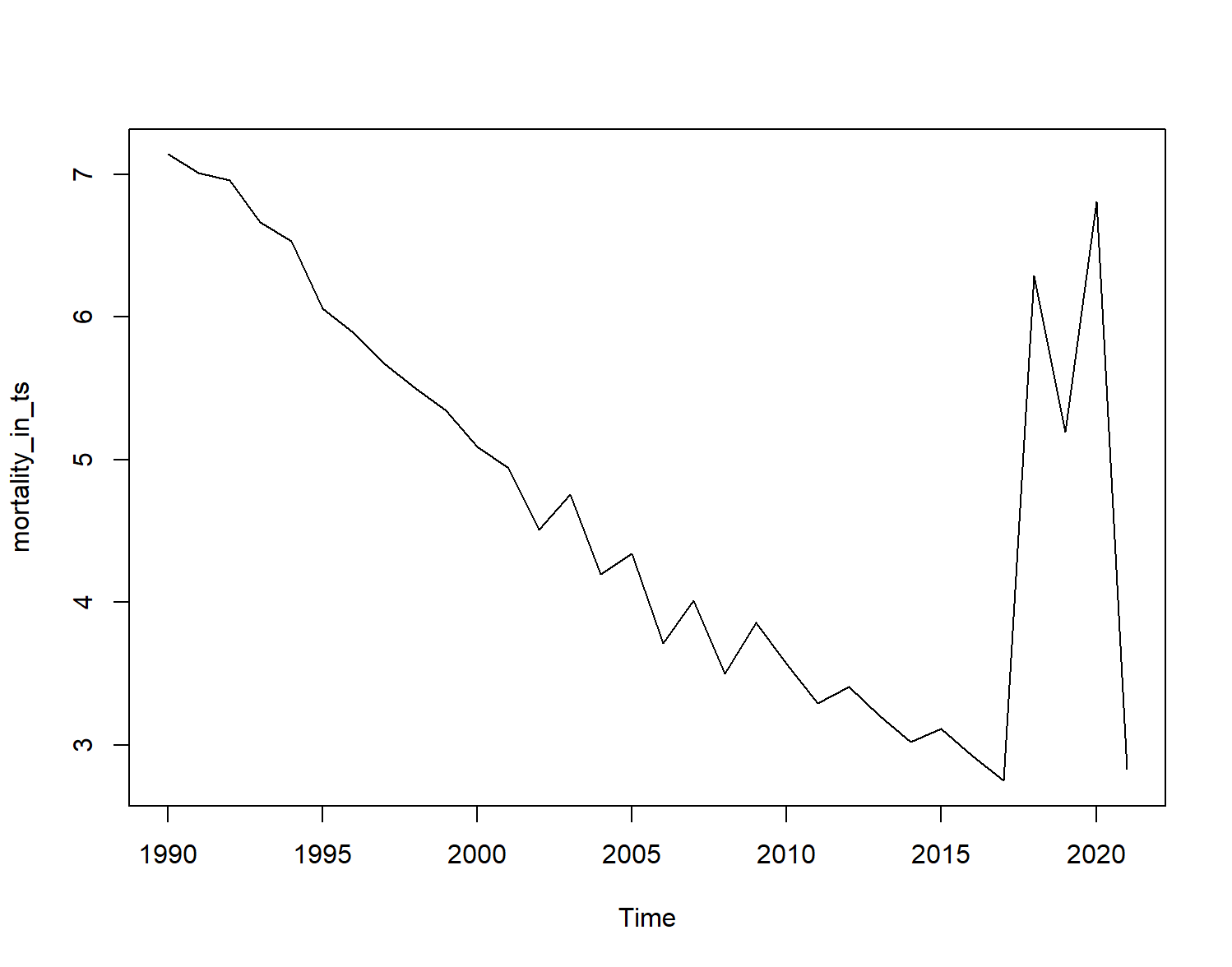
**

**Supplementary Figure 1.** Timing diagram of PU incidence、prevalence and mortality

(a: PU incidence rate;b: PU prevalence rate ;c: PU mortality rate)

a : Incidence


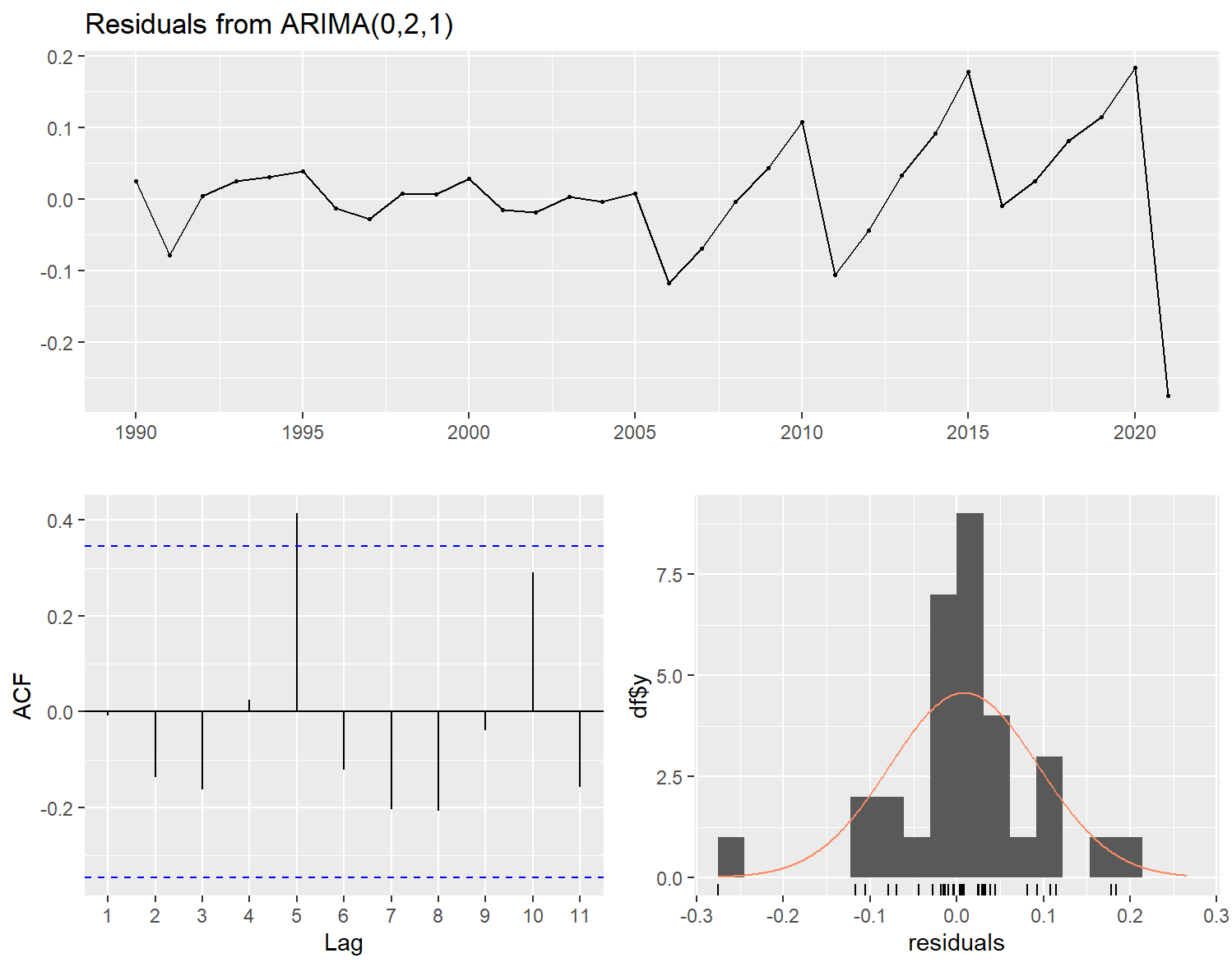

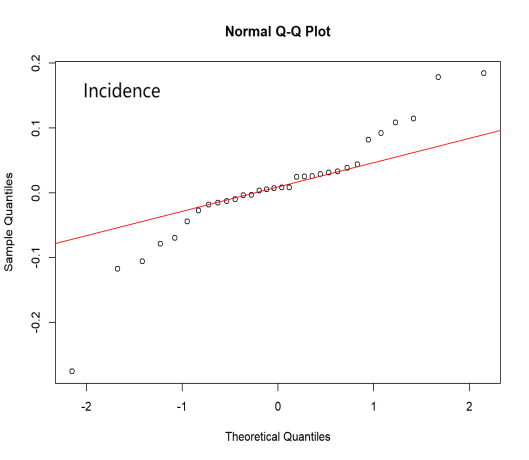


b: Prevalence


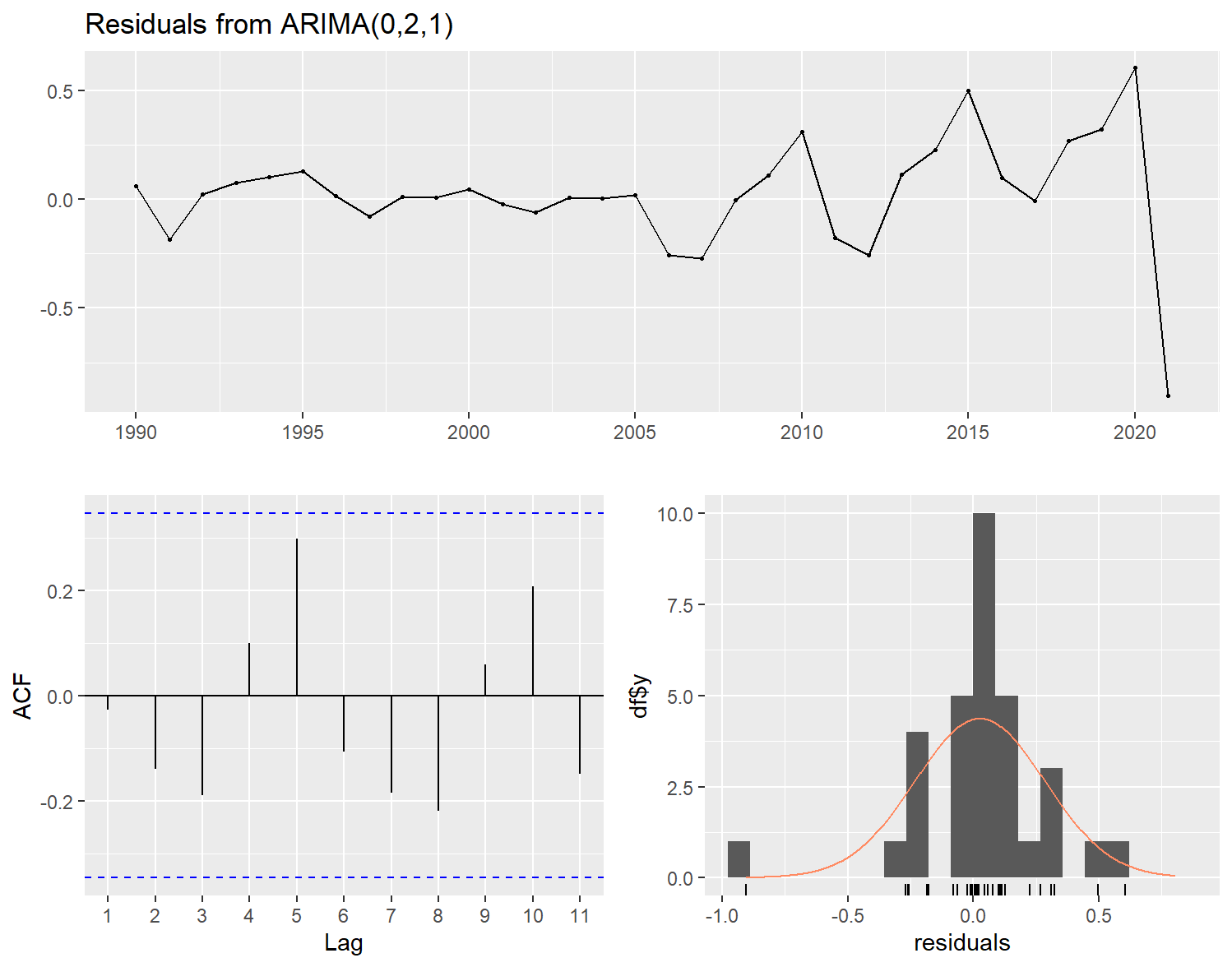

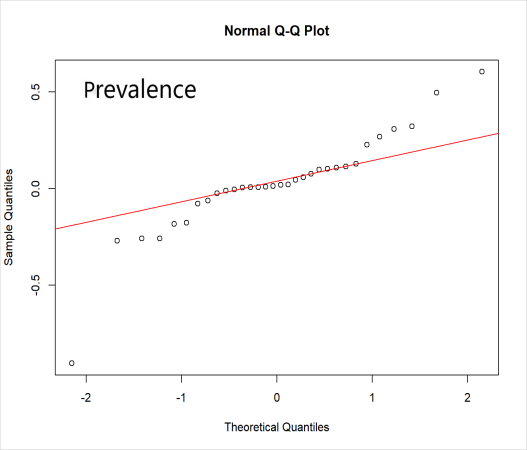


c: Mortality rate


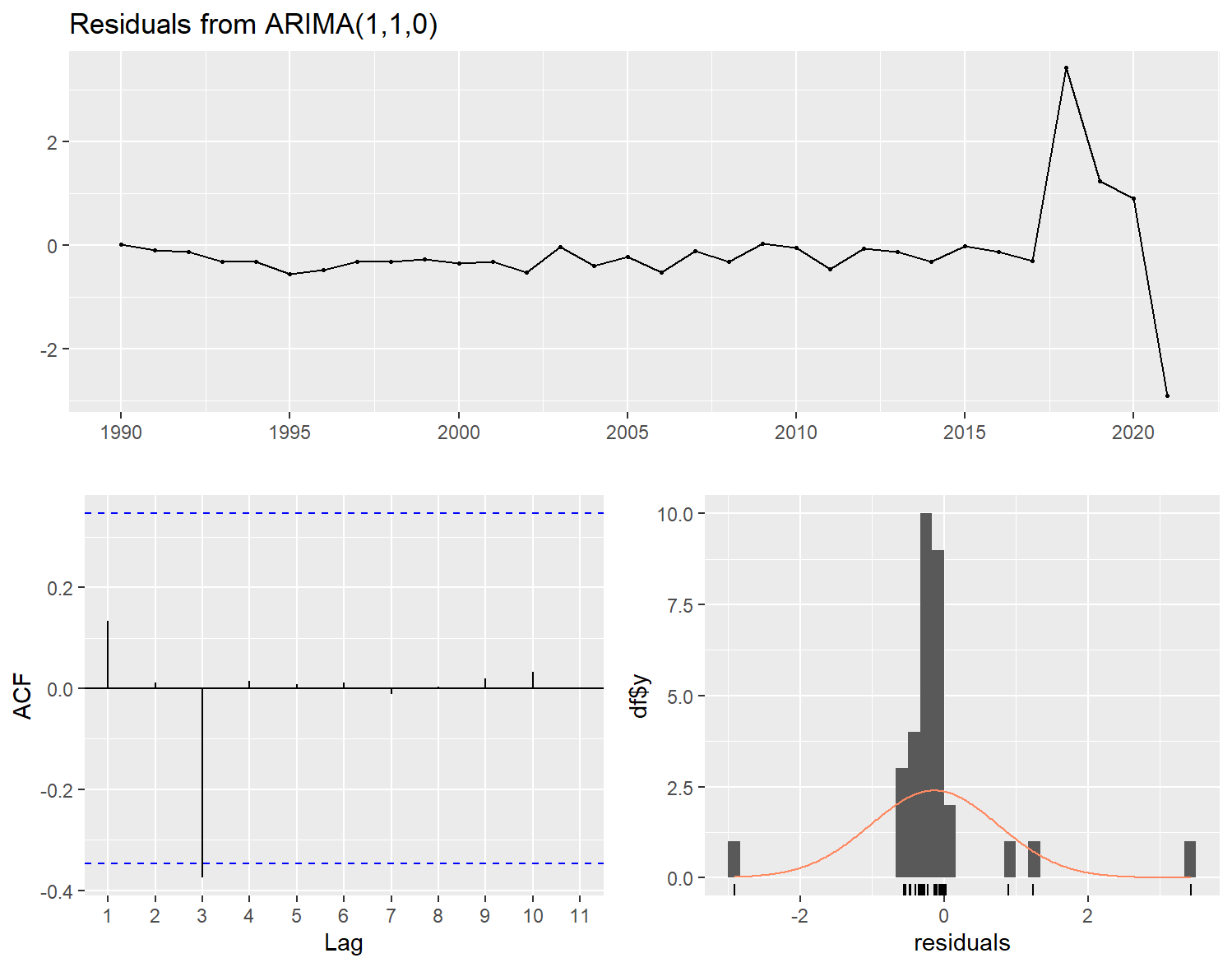

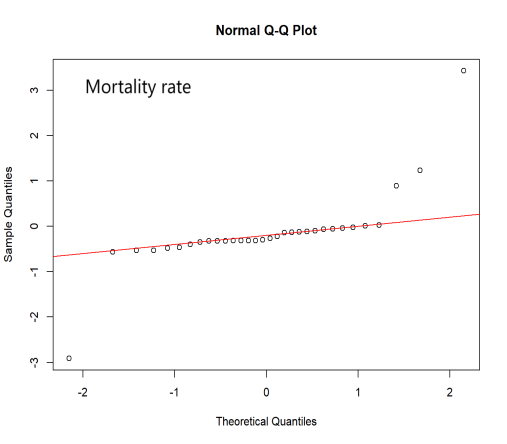


**Supplementary Figure 2.**Residual plots, autocorrelation function (ACF), Ljung-Box test, and residual Q-Q plots of the ARIMA models.(a-c：represent incidence, prevalence, and mortality rate)
